# Nursing training practices. NIC interventions towards healthy aging

**DOI:** 10.15649/cuidarte.4585

**Published:** 2026-04-09

**Authors:** Mónica Margarita Barón Castro, Angelica María Montoya García, María Nelcy Muñoz Astudillo, Yogel Alberto Rúas Amaya

**Affiliations:** 1 Fundación Universitaria del Área Andina, Pereira, Colombia. E-mail: mbaron@areandina.edu.co Fundación Universitaria del Área Andina Pereira Colombia mbaron@areandina.edu.co; 2 Fundación Universitaria del Área Andina, Pereira, Colombia. E-mail: amontoyag@areandina.edu.co Fundación Universitaria del Área Andina Pereira Colombia amontoyag@areandina.edu.co; 3 Fundación Universitaria del Área Andina, Pereira, Colombia. E-mail: marianelcy@gmail.com Fundación Universitaria del Área Andina Pereira Colombia marianelcy@gmail.com; 4 Fundación Universitaria del Área Andina, Pereira, Colombia. E-mail: yruas@areandina.edu.co Fundación Universitaria del Área Andina Pereira Colombia yruas@areandina.edu.co

**Keywords:** Education Nursing, Standardized Nursing Terminology, Professional Training, Adult Health, Healthy Aging, Educación en Enfermería, Terminología Normalizada de Enfermería, Formación Profesional, Salud del Adulto, Envejecimiento Saludable, Educação em Enfermagem, Terminologia Padronizada em Enfermagem, Formação Profissional, Saúde do Adulto, Envelhecimento Saudável

## Abstract

**Introduction:**

With the increasing aging of the population, nursing training must address disability profiles in adults.

**Objective:**

To identify whether Nursing Interventions Classification (NIC) with a population over 29 years of age, carried out by a University Program during intramural practices in hospital institutions, respond to health care guidelines for healthy aging in Colombia.

**Materials and Methods:**

A quantitative cross-sectional descriptive study with an analytical component. Purposive sampling was used. A database containing 13,368 NIC records performed between 2021 and 2023 that met inclusion criteria was analyzed. The population served was characterized; intervention frequencies were obtained according to Functional Health Patterns (FHPs). Using an 80% discrimination standard, five FHPs were included, covering 87.6% (11,714) of the records. Diagnoses and interventions were identified by domains and interpreted according to Colombia's Aging Policy. SPSS-v.26 was used.

**Results:**

The population between 29 and 98 years old was attended, with similar proportions between adults: 50.47% (1,225) and older adults: 49.63% (1,206); male predominance: 62.42% (1,517). The highlighted PFS were Activity and exercise: 33.12% (4,428) and Nutritional-metabolic: 24.17% (2,830), and NIC predominated in the complex physiological domain: 39.29% (4,603): medication administration 48.97% (2,254) and in the basic physiological domain 25.99% (3,045): elimination management 24.86% (757).

**Discussion:**

Interventions are aligned with the goals of wellness, quality of life and autonomy for healthy aging.

**Conclusion:**

The University Institution fulfills its function of training Nursing professionals who respond to the needs of the social environment framed by Public Policies.

## Resumen

**Introducción:**

Con el creciente envejecimiento de la población, la formación de enfermeros debe abordar perfiles de discapacidad en adultos.

**Objetivo:**

Identificar si las intervenciones de Enfermería (NIC) con población mayor de 29 años, realizadas por un programa universitario durante las prácticas intramurales en instituciones hospitalarias, responden a las directrices de atención en salud para el envejecimiento saludable en Colombia.

**Materiales y métodos:**

Estudio descriptivo transversal cuantitativo, con componente analítico. Muestreo intencional. Se analizó una base de datos con 13.368 registros de NIC realizadas entre 2021 y 2023, que cumplieron criterios de inclusión. Se caracterizó la población atendida; se obtuvieron frecuencias de Intervenciones según Patrones Funcionales de Salud (PFS). Con pauta de discriminación del 80% quedaron incluidos cinco PFS abarcando el 87,6% (11.714) de los registros; se identificaron diagnósticos e intervenciones por Dominios que se interpretaron según Política de Envejecimiento en Colombia. Se utilizó SPSS-v.26.

**Resultados:**

Se atendió población entre 29 y 98 años, con proporciones similares entre adultos: 50,47% (1.225) y adultos mayores: 49,63% (1.206); predominio masculino: 62,42% (1.517). Los PFS destacados fueron Actividad y ejercicio: 33,12% (4.428) y Nutricional-metabólico: 24,17% (2.830) y, predominaron NIC en dominio fisiológico complejo: 39,29% (4.603): administración de medicación 48,97% (2.254) y en dominio fisiológico básico 25,99% (3.045): manejo de eliminación 24,86% (757).

**Discusión:**

Las intervenciones se alinean con las metas de bienestar, calidad de vida y autonomía para un envejecimiento saludable.

**Conclusiones:**

La Institución Universitaria cumple su función de formar profesionales de Enfermería que responden a necesidades del medio social enmarcadas en Políticas Públicas.

## Resumo

**Introdução:**

Com o crescente envelhecimento da população, a formação em enfermagem deve abordar os perfis de incapacidade em adultos.

**Objetivo:**

Identificar se as intervenções de Enfermagem (NIC) com uma população acima de 29 anos, realizadas por um programa universitário durante práticas de internação em instituições hospitalares, atendem às diretrizes de saúde para o envelhecimento saudável na Colômbia.

**Materiais e métodos:**

Estudo quantitativo, transversal, descritivo, com componente analítico. Amostragem intencional. Foi analisado um banco de dados com 13.368 registros de NIC realizados entre 2021 e 2023 que atenderam aos critérios de inclusão. A população atendida foi caracterizada; as frequências das intervenções foram obtidas de acordo com os Padrões Funcionais de Saúde (PSF). Utilizando um padrão de discriminação de 80%, cinco PSF foram incluídos, abrangendo 87,6% (11.714) dos registros. Diagnósticos e intervenções foram identificados por Domínios, que foram interpretados de acordo com a Política do Envelhecimento na Colômbia. Utilizou-se o SPSS-v.26.

**Resultados:**

Foi atendida a população entre 29 e 98 anos, com proporções semelhantes entre adultos: 50,47% (1.225) e idosos: 49,63% (1.206); predomínio do sexo masculino: 62,42% (1.517). As EFP destacadas foram Atividade e exercício: 33,12% (4.428) e Nutricional-metabólica: 24,17% (2.830), sendo que a NIC predominou no domínio fisiológico complexo: 39,29% (4.603): administração de medicamentos 48,97% (2.254) e no domínio fisiológico básico 25,99% (3.045): manejo da eliminação 24,86% (757).

**Discussão:**

As intervenções estão alinhadas aos objetivos de bem-estar, qualidade de vida e autonomia para um envelhecimento saudável.

**Conclusões:**

A Instituição Universitária cumpre sua função de formar profissionais de Enfermagem que respondam às necessidades do meio social enquadradas pelas Políticas Públicas.

## Introduction

In 2022, the World Health Organization (WHO) predicted that by 2050, the proportion of people over 60 years of age globally will double, from 12% to 22%, and 80% of these people will live in low-and middle-income countries. Considering that aging directly affects the well-being of older adults, countries must respond with care and social assistance models according to the rate of population aging and the progressive increase in years lived with disability, among the population aged 80 and over^1^,^2^. The WHO proposes the Decade of Healthy Aging (2021-2030) and in its Action Plan calls for the economic, social, political, educational and cultural commitment of all people seeking independence and autonomy for older adults^3^.

Colombia, like the rest of the world, is an aging country. The population over 60 years old increased from 7.5% in 1995 to 15.2% in 2024. The departments with the highest proportion of older people are Quindío: 19.7%, Caldas: 19.3% and Risaralda: 18.4%. These departments have the highest dependency rates for people over 60 years old: Quindío: 31.72%, Caldas: 31.44% and Risaralda, 29.80%^4^ . Changes in population structure and the resulting regulations require healthcare training institutions to understand the social phenomena involved and anticipate short- and medium-term challenges in providing care to adults and older adults.

In defense of the protection of Human Rights of Older Persons, Colombia ratified the provisions of the Inter-American Convention of the Organization of American States (OAS) in June 2015^5^, decreed the Ten-Year Public Health Plan 2022-2031 which included the goal of promoting the functional capacity of the population over 60 years of age^6^. ALikewise, with Decree 681 of 2022^7^ The National Public Policy on Aging and Old Age 2022-2031 was adopted. Currently in Colombia, aging is seen as a fundamental right, as the right to life and dignity in old age; its attention should emphasize the promotion and social protection for care and social assistance, impacting quality of life and well-being^8^. Among the strategies for Comprehensive Health Care with a differential approach is the Promotion and Protection of Older Persons from the Comprehensive Care Route for the Promotion and Maintenance of Health^9^.

These guidelines aim to ensure healthy aging, a dignified, autonomous, and independent old age. Current evidence highlights the importance of nursing's role in active and healthy aging policies and emphasizes the need to adopt a comprehensive, life-course perspective in nursing education professional^10^.

Nurses, as providers of care and health educators par excellence, learn during their training about the care of older people with chronic conditions and progressive deterioration that require long-term management and continuous care; it is precisely in these situations when the rigorous implementation of the Nursing Process becomes crucial, becoming a fundamental tool in the development of competencies for the comprehensive and integrated care of human needs at both the individual and collective levels^11^. The use of the Nursing Process in professional training using standardized language allows for the administration of individualized and patient-centered care while ensuring continuity and consistency of care, favors the teaching-learning process and integra-tes the development of critical and reflective thinking^12^.

The NANDA (North American Nursing Diagnosis Association)-NOC (Nursing Outcomes Classifica-tion)-NIC taxonomy has been standardized internationally. NANDA International Taxonomy II (NANDA-I) categorizes nursing diagnoses into domains and classes, and the latest edition (13th ed.).

NANDA-I 2024–2026, uses Marjory Gordon 's Functional Health Patterns (FHPs)^13^. This manuscript will use the term “Functional Health Patterns” (FHP) and seeks to draw attention to the functional dimensions that structure the comprehensive assessment of the human being, without personalizing the approach with emphasis on the author. The classification of nursing interventions with the NIC taxonomy seeks the standardization and systematization of care in its 8th Edition (2024), the NIC taxonomy has registered 610 intervention labels that are grouped into 7 Domains: 1: Basic Physiological, 2: Complex Physiological, 3: Behavioral, 4: Safety, 5: Family, 6: Health system and 7: Community^14^.

Authors propose that the effective integration of NIC in the training of Nurses is emerging as a fundamental pillar for the development of competent, committed and trained professionals for psychosocial care^15^-^16^, hence, at the University Foundation of the Andean Area-Colombia, since 2011, the Training Practices Information System F_PAE was created and registered nationally, which includes variables of the population served, Teaching-Service Relationship and Nursing Care Processes. This software was created with the NANDA-NOC-NIC taxonomy and is fed by the permanent record of care activities of students and teachers. This study analyzes the Nursing Interventions (NIC) conducted between 2021 and 2023 in the population over 29 years of age during hospital practices of a university program, to evaluate their alignment with the Colombian guidelines for healthy aging. The outcomes will explore how students integrate diagnoses, interventions, and learning with social needs and demographic changes^17^, with the goal of preparing future nurses to face the challenges of chronic disease prevention and comprehensive care for hospitalized adults and older adults, thus contributing to improving the quality of life of these population groups.

## Materials and Methods

A descriptive, cross-sectional, quantitative study with an analytical component was conducted, based on the secondary analysis of data from the F_PAE institutional system (F_PAE1361245/30-06-2017)^18^, registered with the Ministry of the Interior of Colombia. This system, developed by the Andean Area University Foundation, collects information on the service of the population, the teaching-service relationship, and nursing care processes during students' clinical practices.

**Population and sample**

Of a total of 28,664 records corresponding to the 2021-2023 period, 13,368 records were intentionally selected that met the following inclusion criteria: (1) complete and correctly completed registration; (2) individuals in the adult and old age life courses (≥29 years); (3) interventions carried out in hospital institutions within the framework of teaching-service agreements. Records from the community area were excluded due to underreporting caused by health restrictions during the COVID-19 pandemic.

**Ethical considerations**

The Ethics Committee of the Andean Area University Foundation (Minutes of July 27, 2022) approved the study. Access to the database was exclusive to the research team. All records were anonymized and coded to preserve confidentiality.

**Variables and data analysis**

The population was characterized by age, sex, insurance status, socioeconomic status, and type of clinical practice. The relationships between sociodemographic variables and functional health patterns (FHP) were explored using the chi-square test, with a statistical significance level of p<0.05. IBM SPSS Statistics v.26 software was used for the analysis.

For the analysis of nursing interventions, frequencies were identified by PFS. An 80% discrimination standard was applied to ensure that the categories represent consistent and statistically relevant patterns within the sample, aligning with quantitative standards of representativeness and validity^19^, selecting five PFS that concentrated 87.6% (n=11,714) of the total records. In each PFS, the prevalent nursing diagnoses were determined, and the interventions were classified according to the seven domains established by the NIC taxonomy (8th edition, 2024).

The results were interpreted considering the National Policy on Aging and Old Age, allowing for an assessment of the correspondence between the interventions implemented and the guidelines for healthy aging. The complete data set analyzed is available in open access through the Papyrus Dataset repository^20^.

## Results

**Sociodemographic characteristics**

**Figure 1:**
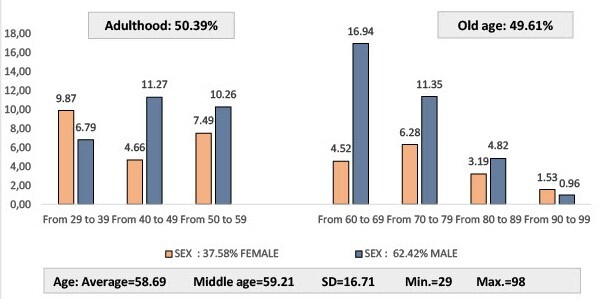
Distribution of the population served in training practices, according to age and sex.

From 2021 to 2023, during the internships carried out by students of the Areandina Nursing Program in different health institutions, 13,368 NIC interventions were recorded in the Institutional Information System on 2,431 people aged 29 and over. As can be seen in Figure 1, the proportion of people by life course was similar: Adulthood: 50.37 % (1,225) and Old Age: 49.63 % (1,206). While among adults (up to 64 years of age) the female population between 30 and 39 years old predominated, in older adults the proportion of the male population was higher, mainly in the 60 to 69 age group.

Regarding health insurance, the largest proportion of the population corresponded to the subsidized regime: 64.93% (1,579). People living in strata 1, 2, and 3 accounted for 97.12% (12,983) of the total study population. The types of practices that recorded the highest proportions of people served were Scientific Principles of Care: 57.09% (1,388), Nursing Care for Adults and Families I: 20.61% (501), and Nursing Care for Adults and Families II: 14.22% (346); the remainder: 8.10% (197) corresponded to other healthcare practices. Table 1 presents the significant relationships between sociodemographic variables and all PFS.

**Table 1:** Significant relationships between Functional Health Patterns and sociodemographic variables of the adult and elderly population served.

Functional Health Pattern	Sociodemographic Characteristics	p-value	Chi square
PFS.1-Perception of Health Maintenance	Sex	0.001	26.18
PFS.1-Perception of Health Maintenance	Social security system	0.001	43.17
PFS.1-Perception of Health Maintenance	SE housing stratum	0.001	87.14
PPFS.1-Perception of Health Maintenance	Age Group	0.001	101.24
PFS.2-Nutritional - metabolic	Sex	0.001	105.38
PFS.2-Nutritional - metabolic	Age Group	0.001	489.13
PFS.3-Elimination	Sex	0.001	338.17
PFS.3-Elimination	SE housing stratum	0.001	100.89
PFS.3-Elimination	Age Group	0.001	1423.96
PFS.4 Activity - exercise	Social security system	0.001	46.56
PFS.4 Activity - exercise	Age Group	0.001	341.84
PFS.5 Rest - Sleep	Social security system	0.017	8.16
PFS.5 Rest - Sleep	SE housing stratum	0.010	14.99
PFS.5 Rest - Sleep	Age Group	0.001	44.02
PFS.6 Cognitive perceptual	Social security system	0.028	7.17
PFS.6 Cognitive perceptual	Sex	0.001	26.40
PFS.6 Cognitive perceptual	SE housing stratum	0.001	21.69
PFS.6 Cognitive perceptual	Age Group	0.001	114.69
PFS.7 Self-image - Self-concept	Social security system	0.001	77.97
PFS.7 Self-image - Self-concept	SE housing stratum	0.001	24.18
PFS.7 Self-image - Self-concept	Age Group	0.001	116.63
PFS.8 Role Relationships	Social security system	0.001	40.30
PFS.8 Role Relationships	SE housing stratum	0.001	37.87
PFS.8 Role Relationships	Age Group	0.001	53.09
PFS.9 Sexuality - Reproduction	Sex	0.001	67.29
PFS.9 Sexuality - Reproduction	SE housing stratum	0.001	40.03
PFS.9 Sexuality - Reproduction	Age Group	0.001	162.62
PFS.10 Coping - Stress Tolerance	Sex	0.001	11.85
PFS.10 Coping - Stress Tolerance	Social security system	0.010	9.29
PFS.10 Coping - Stress Tolerance	Age Group	0.001	152.84
PFS.11 Values - Beliefs	Social security system	0.002	12.97
PFS.11 Values - Beliefs	SE housing stratum	0.001	68.85
PFS.11 Values - Beliefs	Age Group	0.003	21.28

Sources: Nursing Training Practice Information System. Andean Area University Foundation - Pereira and author calculation

As can be seen, the sociodemographic variables: age group (adulthood or old age), sex (female or male), health insurance regime (contributory or subsidized) and housing stratum (low-medium) significantly influence the impact of functional patterns in the population analyzed. 

**Distribution of Functional Health Patterns**

A predominance of five patterns was observed, which covered 87.60% (11,714) of the interventions: Activity/exercise: 33.12% (4,428), Nutritional metabolic: 21.17% (2,830), Elimination: 16.15% (2,159), Perception Health Management: 9.45% (1,263) and Cognitive/perceptive: 7.73% (1,034). In the six remaining PFS only 12.37% (1,654) of the total interventions were recorded. 

Table 2 relates some characteristics of the population served to the relevant PFS. For all PFS, it was common to find that most patients were male, from Risaralda, living in low-income housing, and served by the subsidized health system. 

**Table 2:** Relevant functional health patterns according to the characteristics of the population served

Characteristics	Functional Health Patterns					
	PFS4 Activity/Exercise % (n=815)	PFS2 Nutritional Metabolic % (n=515)	PFS3 Elimination % (n=393)	PFS1 Perception Management Health % (n=230)	PFS6 Cognitive/ perceptual % (n=188)	Rest of PFS % (n=301)
**Life course**						
Adulthood	47.02 (378)	64.95 (334)	30.25 (119)	40.30 (93)	60.64 (114)	62.05 (187)
Old age	52.98 (427)	35.05 (181)	69.75 (274)	59.70 (137)	39.36 (74)	37.95 (114)
**Age group**						
From 29 a 39	13.37 (108)	28.23 (145)	6.76 (27)	16.39 (38)	14.89 (28)	19.96 (60)
From 40 a 49	15.22 (123)	17.17 (88)	7.92 (31)	12.27 (28)	24.08 (45)	23.85 (72)
From 50 a 59	18.43 (148)	19.54 (101)	15.56 (61)	11.64 (27)	21.66 (41)	18.00 (54)
From 60 a 69	16.15 (130)	13.22 (68)	51.18 (201)	23.28 (53)	14.41 (27)	14.07 (42)
From 70 a 79	24.32 (196)	12.93 (67)	10.75 (42)	21.46 (49)	13.64 (26)	16.30 (49)
From 80 a 89	9.42 (76)	7.39 (38)	6.21 (24)	9.90 (23)	6.87 (13)	7.00 (21)
From 90 a 99	3.09 (25)	1.52 (8)	1.62 (6)	5.07 (12)	4.45 (8)	0.54 (2)
**Sex**						
Femele	37.38 (301)	45.90 (236)	20.01 (79)	44.26 (102)	45.07 (85)	37.02 (111)
Male	62.62 (504)	54.10 (278)	79.99 (314)	55.74 (128)	54.93 (103)	62.82 (189)
**Regime**						
Contributory	32.72 (263)	34.17 (176)	34.41 (135)	26.68 (61)	35.59 (67)	34.7 (104)
Subsidized	66.53 (536)	63.96 (329)	64.01 (251)	69.83 (160)	61.80 (116)	61.8 (186)
Bound	0.75 (6)	1.87 (10)	1.57 (6)	3.48 (8)	2.61 (5)	3.5 (10)
**Social stratum**	76.20 (613)	75.37 (388)	75.73 (297)	76.88 (177)	80.56 (151)	73.4 (221)
Low [1 y 2]	76.20 (613)	75.37 (388)	75.73 (297)	76.88 (177)	80.56 (151)	73.4 (221)
Medium [3 y 4]	23.40 (188)	24.20 (125)	24.13 (95)	22.64 (52)	19.15 (36)	26.3 (79)
High [5 y 6]	0.41 (3)	0.42 (2)	0.14 (1)	0.48 (1)	0.29 (1)	0.3 (1)
**Origin**						
Risaralda	86.11 (693)	83.64 (430)	90.37 (355)	92.24 (212)	85.88 (161)	87.1 (262)
Other departments	13.89 (112)	16.36 (84)	9.63 (38)	7.76 (18)	14.12 (27)	12.9 (39)

PFS: Functional Patterns of Health

The PFS Elimination, Perceived health management, and Activity/exercise predominate in older adults, while the Nutritional, Metabolic, and Cognitive-Perceptual PFS are more frequent in adults under 65 years of age. Analysis of trends in PFS with respect to age groups showed that PFS.4 Activity and exercise is the most prevalent, with an upward trend with age, reaching its peak in the 70-79 age group (24.32%). PFS.2 Nutrition shows a downward trend with age, with a higher prevalence in young adults (28.23% in 30-39 age groups), gradually decreasing until reaching its lowest point in 90-99 age groups (1.52%). PFS.3 Elimination varies with age, has a significant peak in the 60-69 age group (51.18%), and is lower in young adults. The PFS.1 Health Perception-Management scores peak in the 60-69 age group and decline with increasing age; similarly, the PFS.6 Cognitive Perceptual Score scores peak in the 40-49 age group (24.08%) and trend downward with advancing age. 

In general, it is observed that activity/exercise and elimination problems are the most prevalent, there is a clear age-dependent relationship in several patterns, nutrition shows an inverse trend with age. 

Correlation analysis showed strong negative co-occurrences between relevant PFS, namely: Activity/ Exercise and Nutrition (-0.36), Activity /Exercise and Elimination (-0.31), Activity/Exercise and Health Perception-Management (-0.23). These negative correlations suggest that when one pattern is present, other patterns are less likely to be present; if an individual seeks interventions for activity/ exercise problems, they are less likely to report simultaneous interventions for nutrition problems; interventions for elimination problems tend not to co-occur with activity/exercise problems. 

**NANDA Diagnostics**

Table 3 presents the distribution of the most frequent NANDA diagnoses by PFS (discrimination cri-terion: 80%). While 10 diagnoses were included in the Activity/Exercise and Nutritional/Metabolic PFS, 80% of the other patterns were represented by five or six more frequent diagnoses. The profile of Nursing diagnoses reveals that during training practices the emphasis is on minimizing the real reason for the patients' complaints, seeking better health and well-being conditions. 

**Table 3:** Nursing Diagnoses [NANDA I]* according to relevant Functional Health Patterns identified during training practices.

Relevant Functional Health Patterns Records 100% (n=11,714)	Nursing Diagnoses [NANDA I] (Discrimination= 80%)	% (n)**
4. Activity/Exercise. Records: 37.80 (4,428)	0032. Ineffective breathing pattern 0029. Decreased cardiac output 0085. Impaired physical mobility 0030. Impaired gas exchange 0100. Delayed surgical recovery 0093. Fatigue 0298. Decreased activity tolerance 0204. Ineffective peripheral tissue perfusion 0182. Willingness to improve self-care 0033. Impaired spontaneous ventilation Functional pattern subtotal Rest NANDA Pattern Activity/Exercise	17.55 (777) 14.86 (658) 13.39 (593) 8.90 (394) 6.73 (298) 4.95 (219) 4.67 (207) 3.97 (176) 3.18 (141) 2.19 ( 97) 80.40 (3560) 19.60 ( 868)
2. Nutritional Metabolic Records: 24.16 (2,830)	0044. Impaired tissue integrity 0047. Impaired skin integrity 0103. Impaired swallowing 0045. Impairment of the integrity of the oral mucosa 0026. Excess fluid volume 0027. Fluid volume deficit 0179. Risk of unstable blood glucose level 0266. Risk of surgical wound infection 0312. Pressure injury in adults 0248. Risk of deterioration of the integrity of the oral mucosa Functional pattern subtotal Rest NANDA Nutritional/Metabolic Pattern	20.85 (590) 20.46 (579) 9.22 (261) 7.92 (224) 5.87 (166) 4.73 (134) 4.06 (115) 2.83 ( 80) 2.61 ( 74) 2.05 ( 58) 80.60 (2281) 19.40 (549)
3. Elimination Records: 18.43 (2,159)	0016. Impaired urinary elimination 0023. Urinary retention 0022. Risk of urge urinary incontinence 0322. Risk of urinary retention Functional pattern subtotal Remainder NANDA Pattern Elimination	42.70 (922) 21.21 (458) 14.54 (314) 6.95 (150) 85.41 (1844) 14.58 (315)
1. Perception of Health Management Records 10.78 (1,263)	0257. Elderly frailty syndrome 0276. Ineffective self-management of health 0043. Ineffective protection 0266. Risk of infection 0293. Willingness to improve self-management of health 0292. Ineffective health maintenance behaviors Functional pattern subtotal Rest NANDA P. Perception Health Management	21.62 (273) 15.52 (196) 15.28 (193) 15.20 (192) 10.21 (129) 8.16 (103) 85.99 (1086) 14.01 (177)
6. Cognitive/Perceptual Records 8.83 (1,034)	0132. Acute pain 0214. Discomfort 0133. Chronic pain 0131. Memory impairment 0279. Altered thought process Functional pattern subtotal Rest NANDA Cognitive-Perceptual Pattern	41.49 (429) 17.31 (179) 13.06 (135) 5.90 ( 61) 5.90 ( 61) 83.70 (865) 16.30 (169)

*Source: Nursing Diagnoses: Definitions and Classification 2021-2023. 12th ed. ** Note: % = percentage in columns and n = number of records per PFS.

**NIC Interventions**

Table 4 presents the frequency of nursing interventions by intervention domain. In the complex physiological domain, medication administration is highlighted, followed by airway management; these should be the nursing professional's greatest strengths in the clinical area. In the basic physiological domain, according to the diagnostic profile, urinary elimination management and assistance with self-care are highlighted. In the behavioral domain, teaching and patient agreement predominate, and in the safety domain, infection control and skin surveillance predominate. NIC interventions during nursing training are fully articulated with the PFS and nursing diagnoses relevant to the adult and older adult population. 

**Table 4:** Nursing Interventions (NIC) according to Domain

NIC Domains Registrations 100% (n=1,714)	Nursing Interventions (NIC)	% (n)*
Complex Physiological Records: 39.29 (4,603)	Medication administration Airway management/Monitoring/Oxygen therapy Cardiac Care: Rehabilitation/Acute/Arrhythmia Wound/skin/access care/monitoring Fluid Management - Electrolytes/Monitoring/Dialysis Circulatory care: venous/arterial insufficiency NIC remainder complex physiological domain	48.97 (254) 13.62 (627) 13.12 (604) 7.63 (351) 6.52 (300) 4.24 (195) 5.91 (272)
Basic Physiological Records: 25.99 (3,045)	Urinary elimination management/ retention care/ incontinence care Help with self-care Nutrition Management Exercise promotion/ Weight loss aid Acute/Chronic/Positional Pain Management Exercise/activity/relaxation therapy Maintenance/Restoration of oral health Rest NIC basic physiological domain	24.86 (757) 17.50 (533) 15.96 (486) 12.15 (370) 13.50 (411) 6.67 (203) 5.91 (180) 3.09 ( 94)
Behavioral Records: 19.04%(2,230)	Teaching: individual/: medications /: disease process/ Health education Emotional support/Mood management Patient Agreement/Spiritual Support Sexual counseling Decreased anxiety Help in self-modification Cognitive stimulation Rest NIC behavioral domain	29.19 (651) 19.82 (442) 18.52 (413) 12.56 (280) 8.16 (182) 3.32 (74) 1.97 (44) 6.46 (144)
Security Records: 7.52%(881)	Infection control/Infection protection Risk identification Surveillance Isolation/Validation Therapy Triage: emergency center Dementia management Rest NIC domain security	54.60 (481) 18.16 (160) 8.17 (72) 7.26 (64) 6.58 (58) 1.14 (10) 4.08 (36)
Health System Records: 4.40% (516)	Orientation in the health system Interpretation of laboratory data Sample handling Health care information exchange Discharge Planning Rest NIC domain Health System	26.74 (138) 21.32 (110) 13.95 (72) 13.95 (72) 13.18 (68) 10.85 (56)
Family Records: 3.75% (439)	Promote family involvement Maintaining family processes Stimulation of family integrity Family therapy Family mobilization	51.48 (226) 20.50 (90) 20.50 (90) 7.29 (32) 5.47 (24)

*Nota: % = porcentaje en columnas y n= número de registros por Dominio NIC

## Discussion

In line with the objective of this study, the indicators from the National Observatory on Aging and Old Age show that, in Risaralda, in 2022, the morbidity treated in people over 60 years of age was mainly due to diseases of the circulatory system and Diabetes, which were more common in women; fo-llowed by diseases of the urinary system, communicable diseases, and the respiratory system, which were more prevalent in men^21^. Consistent with the above, in our study, the highest proportions of interventions were concentrated in Activity /Exercise, Nutritional Metabolism, Elimination, Health Perception/Management, and Cognitive/Perceptual. This result demonstrates that the nursing tra-ining practices in the clinical area at the selected university institution do respond to the regional guidelines for health care during adulthood and old age, in terms of morbidity. The interventions are aimed at minimizing functional dependency and disability and improving the quality of life of older adults, as outlined in the Colombian Policy on Aging and Old Age^8^.

As observed in the frequency of PFS, Nursing Interventions (NIC) for the adult and older adult population, delivered during training practices, correspond to the professional training needs and management guidelines for the most prevalent events in this population. Thus, the largest proportions correspond to medication administration, airway management, urinary elimination management, assistance with self-care, patient education and agreements, i nfection control, and skin surveillance.

Training practices in health allow the integration of theoretical and practical knowledge; this contributes to a more complete humanistic and professional training^22^. These educational experiences, when aligned with NIC interventions, as shown in this study, favor the development of competencies for the comprehensive and integrated care of health/illness experiences, promo-ting ethical and people-centered interventions, which at any time in their lives are oriented towards healthy aging.

Similarly, recent research^23^,^24^ addresses nursing care practices in adults and older adults, highligh-ting the importance of interventions focused on promoting mobility and improving nutritional status to prevent functional decline. Chronic diseases such as Hypertension, Diabetes, and heart disease are commonly associated with decreased physical function in this population^3^. Consistently, during the training practices recorded in this study, a predominance of interventions in the "Activity and exercise" and "Nutrition-metabolic" patterns was observed.

Maintaining regular physical activity not only improves functional capacity but also contributes to the preservation of cognitive and physical reserve in older adults, resulting in greater independence and quality of life^25^. Finding this pattern relevant in this study means that students are acquiring skills to promote physical activity and prevent functional decline, thus contributing to comprehensive patient-centered care.

On the other hand, recent studies with biomarkers^26^ have indicated that socioeconomic conditions throughout life decisively affect health in old age, mainly in the increase of cardiovascular risk. The findings of this study show a population attended for chronic non-communicable diseases of cardiovascular and metabolic type, coming from strata 1 and 2 and with a subsidized insurance regime.

## Conclusions

The predominance of adult and elderly patients treated under the subsidized system from low socioeconomic strata and the high percentage of interventions targeted at them reflect the influ-ence of socioeconomic profile on access to health care. Care strategies emanating from public policies are essential to reducing inequalities and improving the well-being of these individuals.

The analysis of the nursing training practice information system at the selected institution highlights the importance of using PFS in assessing the health status of the populations served. The integration of NANDA Diagnoses and NIC Interventions allows for identifying the competencies nursing students are developing for the comprehensive care of adults and older adults, and in turn, orienting interventions toward healthy aging policies.

The most frequent NIC interventions focused on the PFS of Activity and Exercise, Nutritional-Metabolic, Elimination, Health Perception/Management, and Cognitive/Perceptual; this coincides with the morbidity profile described by the National Observatory on Aging and Old Age, which allows us to conclude that the nursing training practices in the clinical area at the selected university institution do respond to the guidelines for health care during adulthood and old age in the region.

Regarding Nursing Interventions, the following are highlighted, from highest to lowest proportion: Medication Administration, followed by airway management (Complex Physiological), urinary elimination management and assistance with self-care (Basic Physiological), patient education and agreement (Behavioral), infection control and skin surveillance (Safety). This intervention profile shows that Nursing Professionals at the selected Institution are acquiring key competencies required for comprehensive and effective care in the hospital setting, both in highly complex health phenomena and in basic care and health promotion in the adult and elderly population.
